# Urea cycle disorders in Spain: an observational, cross-sectional and multicentric study of 104 cases

**DOI:** 10.1186/s13023-014-0187-4

**Published:** 2014-11-30

**Authors:** Elena Martín-Hernández, Luis Aldámiz-Echevarría, Esperanza Castejón-Ponce, Consuelo Pedrón-Giner, María Luz Couce, Juliana Serrano-Nieto, Guillem Pintos-Morell, Amaya Bélanger-Quintana, Mercedes Martínez-Pardo, María Teresa García-Silva, Pilar Quijada-Fraile, Isidro Vitoria-Miñana, Jaime Dalmau, Rosa A Lama-More, María Amor Bueno-Delgado, Mirella del Toro-Riera, Inmaculada García-Jiménez, Concepción Sierra-Córcoles, Mónica Ruiz-Pons, Luis J Peña-Quintana, Inmaculada Vives-Piñera, Ana Moráis, Elena Balmaseda-Serrano, Silvia Meavilla, Pablo Sanjurjo-Crespo, Celia Pérez-Cerdá

**Affiliations:** Pediatric Rares Diseases Unit, Metabolic and Mitochondrial Diseases, Pediatric Department, Hospital Universitario 12 de Octubre. Research Institute (i +12), Madrid, Spain, Avda de Córdoba s/n, 28041 Madrid, Spain; H.U. de Cruces, Bilbao, Spain; H. Sant Joan de Déu, Barcelona, Spain; H.U. Infantil del Niño Jesús, Madrid, Spain; C.H.U. de Santiago, Santiago de Compostela, Spain; H. Materno Infantil Carlos Haya, Málaga, Spain; H.U. Germans Trias i Pujol, Badalona, Spain; H.U. Ramón y Cajal, Madrid, Spain; H. Infantil La Fe, Valencia, Spain; H.U. La Paz, Madrid, Spain; H.U. Virgen del Rocío, Sevilla, Spain; H. Vall d’Hebrón, Barcelona, Spain; H.U. Miguel Servet, Zaragoza, Spain; C.H. de Jaén, Jaén, Spain; H.U. Ntra. Sra. de la Candelaria, Santa Cruz de Tenerife, Spain; H.U. Materno Infantil de Las Palmas, Las Palmas de Gran Canaria, Spain; H.C.U. Virgen de la Arrixaca, Murcia, Spain; C.H.U. de Albacete, Albacete, Spain; CEDEM. Facultad de Ciencias, Universidad Autónoma de Madrid, Madrid, Spain

**Keywords:** Urea cycle disorders, UCDs, N-acetylglutamate synthase, Carbamoylphosphate synthetase 1, Ornithine transcarbamylase, Argininosuccinate synthetase, Citrullinemia type 1, Argininosuccinate lyase, Argininosuccinic aciduria, Arginase 1

## Abstract

**Background:**

Advances in the diagnosis and treatment of urea cycle disorders (UCDs) have led to a higher survival rate. The purpose of this study is to describe the characteristics of patients with urea cycle disorders in Spain.

**Methods:**

Observational, cross-sectional and multicenter study. Clinical, biochemical and genetic data were collected from patients with UCDs, treated in the metabolic diseases centers in Spain between February 2012 and February 2013, covering the entire Spanish population. Heterozygous mothers of patients with OTC deficiency were only included if they were on treatment due to being symptomatic or having biochemistry abnormalities.

**Results:**

104 patients from 98 families were included. Ornithine transcarbamylase deficiency was the most frequent condition (64.4%) (61.2% female) followed by type 1 citrullinemia (21.1%) and argininosuccinic aciduria (9.6%). Only 13 patients (12.5%) were diagnosed in a pre-symptomatic state. 63% of the cases presented with type intoxication encephalopathy. The median ammonia level at onset was 298 μmol/L (169-615). The genotype of 75 patients is known, with 18 new mutations having been described. During the data collection period four patients died, three of them in the early days of life. The median current age is 9.96 years (5.29-18), with 25 patients over 18 years of age. Anthropometric data, expressed as median and z-score for the Spanish population is shown. 52.5% of the cases present neurological sequelae, which have been linked to the type of disease, neonatal onset, hepatic failure at diagnosis and ammonia values at diagnosis. 93 patients are following a protein restrictive diet, 0.84 g/kg/day (0.67-1.10), 50 are receiving essential amino acid supplements, 0.25 g/kg/day (0.20-0.45), 58 arginine, 156 mg/kg/day (109-305) and 45 citrulline, 150 mg/kg/day (105-199). 65 patients are being treated with drugs: 4 with sodium benzoate, 50 with sodium phenylbutyrate, 10 with both drugs and 1 with carglumic acid.

**Conclusions:**

Studies like this make it possible to analyze the frequency, natural history and clinical practices in the area of rare diseases, with the purpose of knowing the needs of the patients and thus planning their care.

## Background

The urea cycle is the final common pathway for the excretion of waste nitrogen as well as arginine synthesis [1,2]. Urea cycle disorders (UCDs) are caused by a deficiency of one of the six enzymes in this cycle. Three of the enzymes are mitochondrial, N-acetylglutamate synthase (NAGS), carbamoylphosphate synthetase 1 (CPS1), and ornithine transcarbamylase (OTC), and the other three are cytoplasmic, argininosuccinate synthetase (ASS), argininosuccinate lyase (ASL) and arginase 1 (ARG1). As the metabolic steps take place in two different cellular compartments, two transporters are also necessary: the ornithine/citrulline antiporter ORNT1, deficiency of which causes hyperornithinemia-hyperammonemia-homocitrullinuria syndrome and the glutamate/aspartate antiporter CITRIN, deficiency of which gives rise to type 2 citrullinemia. All these deficiencies are inherited in an autosomal recessive manner, except for OTC deficiency which is inherited in an X-linked recessive manner.

The incidence of UCDs varies between 1:22.179-1:53.717 newborns [3-7] although it could be higher, considering that not all the cases are detected by expanded newborn screening (NBS) and many can be undiagnosed cases with a fatal outcome.

The clinical presentation is variable; the onset of severe forms usually occurs during the neonatal period and it is characterized by food refusal, vomiting, lethargy, polypnea, and rapid progression to coma and multiorgan failure [8]. There is high mortality among this type of patients and those who survive experience frequent subsequent decompensation and bad neurological prognosis [9]. The onset of mild forms can occur at any age with hyperammonemic episodes triggered during metabolic stress (infections, vomiting, surgery, etc.) or with more insidious symptoms such as failure to thrive, liver disease, developmental delay, behavioral disorders or psychiatric symptoms [10]. The biochemical data that helps making the diagnosis is hyperammonemia together with the amino acid and organic acid pattern. The diagnosis is confirmed through an enzymatic and/or genetic study. Treatment is primarily based on a low protein diet, essential amino acids (EAA) supplements, arginine and/or citrulline, and the use of nitrogen scavenging drugs such as sodium benzoate (BZ) or sodium phenylbutyrate (PBA) [11]. No protein restriction is necessary when treating NAGS deficiency with carglumic acid.

Diagnostic and treatment advances over recent years, including newborn screening (NBS) for some of the conditions [12,13], acute hyperammonemia treatment guidelines [11,14,15], extracorporeal detoxification [16,17], nutritional therapy [18], nitrogen scavenging drugs [19], liver transplantation [20,21], etc., have decreased the mortality rates, and many patients reach adulthood. However, neurological sequelae are still prominent. Since these are rare conditions and the number of patients in each center is low, it is necessary to conduct multicenter studies to understand the new natural history of the disease. A registry of patients has been started recently in the USA [22] and another one in Europe [23]. In Spain there was no information on patients with these diseases, so the Spanish Association of Inborn Errors of Metabolism (AECOM) has promoted this observational, cross-sectional and multicenter study that aims to find out the number of patients with urea cycle disorders, as well as their clinical and laboratory characteristics, in order to plan their care.

## Methods

Data used in the analysis were collected at enrolment in a national registry of patients with UCDs. The study was carried out by the Spanish urea cycle disorders study group, members of the AECOM. All inherited metabolic diseases (IMD) centers in Spain, covering the entire population of the country, answered a questionnaire regarding clinical and analytical information at diagnosis and at last visit of patients with UCDs alive during the study period.

The inclusion criteria were:

1. Data collection between February 2012 and February 2013, 12 months.
2. Live patients at any time during the study period, even if the patient died later on.
3. Urea cycle disorder diagnosis due to a deficiency of any of the six enzymes of the cycle.
4. Heterozygous mothers of patients with OTC deficiency were only included if they were on treatment due to being symptomatic or having biochemistry anomalies.

Informed consent was obtained for their inclusion in the registry and for anonymous publication of the data. The data were anonymized before their assessment and statistical analysis. The registry was approved by the Ethics Committee of the 12 de Octubre Hospital (Madrid, Spain).

The data obtained at diagnosis was: type of disorder, gender, age at onset of symptoms and age at diagnosis, ethnic origin, UCD family history or family consanguinity, presentation (neonatal, late or pre-symptomatic), diagnostic method (biochemical, enzymatic, genetic), clinical symptoms, biochemical parameters and molecular study. Biochemical and molecular studies were performed over a long period of time in different laboratories using diverse methods, some of them previously published [24-28]. In none of the cases the molecular study was done by next-generation sequencing (NGS).

Data obtained on the last visit during the study period was: age, anthropometric data (weight, height and head circumference), liver damage (hypertransaminasemia, liver hyper-echogenicity), neurological impairment (developmental delay, mental retardation, learning disorder, behavior disorders, motor disorders and epilepsy), biochemical parameters, dietary treatment (total protein, energy, essential amino acids, L-arginine, L-citrulline) and pharmacological treatment. Anthropometric parameters were collected by standard procedures in each center and expressed as z-scores, using published Spanish population values as a reference [29].

Statistical methods: data are expressed as mean and standard deviation (SD) or median and interquartile range (IQR) for quantitative variables and percentages for qualitative variables. The level of statistical significance was obtained using Chi-square or Fisher’s Exact Test for qualitative variables and Student’s *T*-test or the Mann-Whitney *U* test for quantitative variables.

## Results

### Data at diagnosis

#### Demographic data

A total of 104 patients from 98 different families were included, with the following diagnosis: 67 OTC deficiency (64.4%), 22 ASS deficiency (type 1 citrullinemia) (21.1%), 10 ASL deficiency (argininosuccinic aciduria) (9.6%), 2 CPS1 deficiency (1.92%), 2 ARG1 deficiency (1.92%) and 1 NAGS deficiency (0.96%). Some of the patients have been previously published [24-28,30].

Almost half of the patients (47%, 49 cases) were male and 53% (55) female. From the 67 cases with OTC deficiency, 38.8% (26) were male and 61.2% (41) female (Table 1). Other relatives were affected in 20.4% of the cases and there was family consanguinity in 5.9% of the cases. Most patients were European (90.4%, 94 subjects), with a small number of patients from other geographical origins: 5.7% (6) from Morocco, and 3.8% (4) from Latin America.

**Table 1:** **Description of the series**

**Disease**	**Number (%)**	Gender*		Presentation*		
		**Male**	**Female**	**Symptomatic**		**Asymptomatic**
				**Neonatal**	**Late**	
OTCD	67 (64.4)	26 (38.8)	41 (61.2)	9 (13.4) 7 males	52(77.6) 15 males	6 (9) 4 males
ASSD	22 (21.1)	13 (59)	9 (61)	14 (63.6)	4 (18.2)	4 (18.2)
ASLD	10 (9.61)	6 (60)	4 (40)	2 (20)	6 (60)	2 (20)
CPS1D	2 (1.92)	1 (50)	1 (50)	1(50)	1 (50)	
ARG1D	2 (1.92)	2 (100)	0	1 (50)		1 (50)
NAGSD	1 (0.96)	1 (100)	0	-	1 (100)	-
Total	104 (100) (98 families)	49 (47.1)	55 (52.9)	27 (26)	64 (61.5)	13 (12.5)

OTCD: ornithine transcarbamylase deficiency. ASSD: argininosuccinate synthetase deficiency; ASLD: argininosuccinate lyase deficiency; CPS1D: carbamoylphosphate synthetase 1 deficiency; ARG1D: arginase 1 deficiency; NAGSD: N-acetylglutamate synthase deficiency.

*Number of cases (%).

During the study time 11 new cases were diagnosed, 6 of them were born in that period (3 with neonatal onset and 3 diagnosed by NBS) and 5 had been born in previous years.

The median age at onset, in patients with clinical symptoms, was 12.7 months (0.2-28) and the median age at diagnosis was 16.3 months (0.2-43.2). The median age of asymptomatic patients at diagnosis was 0.46 months (0.17-53).

During the first year 44.2% of the cases (46) were diagnosed, between 1 year and 6 years, 39.4% (41), between 6 and 12 years 11.5% (12), between 12 and 18 years 1.9% (2) and the remaining 2.8% (3) were diagnosed in adulthood.

#### Symptoms and biochemical data at diagnosis

Symptoms began during the neonatal period in 25.9% of the cases (27), 61.5% (64) had a late onset and only 12.5% (13) were asymptomatic at diagnosis. Therefore, 87.5% of the patients (91) were index-patients diagnosed due to symptoms. Neurological deterioration “intoxication type” was present in 63% of the cases. The rest of the symptomatic patients presented other neurological and/or digestive symptoms at onset. Psychiatric symptoms, such as psychosis in the 12 year old NAGSD patient [30],were observed in 1.9% of the patients.

Of the 13 cases diagnosed in the pre-symptomatic state, 5 were detected through NBS (3 ASSD, 1 ASLD and 1 ARG1D), one was a prenatal diagnosis (ASL deficiency), and the remaining 7 were diagnosed through family history (6 OTCD and 1 ASSD).

**Figure 1:**
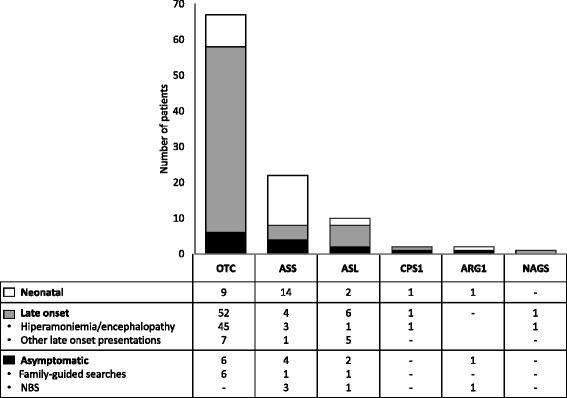
**The table under the figure shows the number of cases with neonatal, late or asymptomatic presentation in each disease.** Most of the ASS deficient patients had neonatal onset. Among asymptomatic patients with OTC deficiency only one was a heterozygous mother included and treated because of biochemical abnormalities.

Figure 1 shows the type of presentation according to the disorder. 63.6% of the patients with type 1 citrullinemia had neonatal onset, while in OTC and ASL deficiencies a late onset was more frequent (77.6% and 60%, respectively). Among patients with OTC deficiency, 9 had neonatal onset (7 males), 52 had late onset (15 males) and 6 were asymptomatic at diagnosis (4 males). 4 of the asymptomatic patients were children (3 males, 1 female); another one was a heterozygous mother treated because she had very low levels of citrulline and arginine at diagnosis and the last one was an adult man with biochemical abnormalities, who was also treated in order to prevent an acute hyperammonemic encephalopathy.

With respect to ARG1 deficiency, a very rare disease, there are two patients in our series. The first one is a male diagnosed through NBS with arginine levels of 112 μmol/L at 48 h of age (reference range: 0.2- 49.54). Ammonia and glutamine were normal (50 and 350 μmol/L respectively) and the diagnosis was confirmed by enzymatic activity in red cells. The second one is a male who presented at 2 days of age with respiratory distress, food refusal and seizures. Ammonia level was 164 μmol/L, glutamine 1105 μmol/L and arginine 590 μmol/L. He was diagnosed by enzymatic study in red cells.

Median ammonia level at onset in symptomatic patients was 298 μmol/L (169-615) and median glutamine level was 1124 μmol/L (859-1546). Three cases with argininosuccinic aciduria and 1 case with OTC deficiency showed normal values of ammonia and glutamine at onset. 17 patients had biochemical parameters consistent with acute liver failure. Other biochemical data are shown in Table 2. Blood levels of citrulline and arginine in each type of disease are shown in Table 3.

**Table 2:** **Biochemical data at diagnosis (excluding asymptomatic patients) and at last visit (all patients)**

Biochemical data *	**At clinical onset (N = 91)**	**At last visit (N = 96)**	**Normal values**
Ammonia (μmol/L)	298 (169-615)	33 (27-42)	10-55 (80**)
Glutamine (μmol/L)	1124 (859-1546)	716 (545-908)	350-650
Prothrombin activity (%)	60 (32.6-82)	91 (85-100)	75-130
ALT (IU/L)	76 (38-159)	24 (16-34)	5-39
AST (IU/L)	53.5 (36.7-161)	27.5 (19-37.2)	5-37
Bilirrubin (mg/dL)	0.60 (0.43-3.77)	0.53 (0.30-0.80)	0.20-1.10
Albumin (g/L)	3.79 (3-4.41)	4.30 (3.90-4.50)	3.80-5.40
pH	7.43 (7.36-7.46)	-	7.35-7.45
Bicarbonate (mmol/L)	21 (19-24)	-	23-27

*Median and interquartile range (IQR); **Normal values in newborns.

**Table 3:** **Levels of specific urea cycle amino acids at diagnosis for the different diseases**

**Type of UCD**	Amino acid*	
	**Citrulline (μmol/L) (NV: 26 ± 8)**	**Arginine (μmol/L) (NV: 64 ± 24)**
NAGSD, CPS1D, OTCD	10 (7-16)	39 (20-59)
ASSD	1654 (984-2400)	31 (17-41)
ASLD	173 (103-256)	36 (24-81)
ARG1D	21 (20.5- 21.5)	351 (231-470)

*Median and interquartile range (IQR).

NV: normal values.

NAGSD: N-acetylglutamate synthase deficiency; CPS1D: Carbamoylphosphate synthetase 1 deficiency; OTCD: Ornithine transcarbamylase deficiency; ASSD: Argininosuccinate synthetase deficiency; ASLD: Argininosuccinate lyase deficiency; ARG1D: Arginase 1deficiency.

The diagnosis was confirmed through enzymatic activity in 40.4% (42) and/or through molecular study in 79.8% (83) of the cases, although we only have available the genotype of 72.1% (75) of the subjects. In 12 (11 symptomatic, 1 asymptomatic) OTC female patients, despite their mosaic nature, deficient OTC activity was supported in jejunal biopsy.

#### Molecular genetic data

The genetic study was available in 79 patients (56 OTC, 14 ASS, 7 ASL, 1 CPS and 1 NAGS). The genotype of 72.1% of the patients (75) is known. Eight OTC, 2 ASS, 5 ASL, 1CPS and 2 NAGS mutations have not been previously reported.

Table 4 lists the 39 different mutations of the 52 patients (48 families) with OTC deficiency. In 4 OTC deficient patients mutations were not found. Therefore the sensitivity of our genetic studies in OTC was 92.85%. Table 4 includes information on the gender, time and severity at onset and neurological situation at last visit. References of previously reported cases are indicated next to the number of the patient [24-27]. The most frequent mutation was p.Arg129His which was identified in 5 patients (3 females) from 4 families, all of them with good neurologic outcome. On the other hand, the two patients with a deletion of the whole OTC gene are very symptomatic. For other frequent mutations, such as p.Arg40His or p.Gly195Arg, neurological outcome is related more to the time and severity at onset rather than the mutation itself, with poorer outcome of index-cases than asymptomatic siblings diagnosed through family history (Table 4).

**Table 4:** **Mutations identified in 52 patients (48 families) with OTC deficiency**

**Patient n°/gender/[reference]**	**Nucleotide**	**Protein**	**Onset**	**Neurologic damage**	**Alive**
**4 M**	c.1028C > G*	p.Thr343Arg*	Late	No	Yes
5 F	c.663 + 2 T > C (IV6 + 2 T > C)		Late	Yes	Yes
7 F	c.533C > T	p.Thr178Met	Late	No	Yes
8 M	c.421C > T	p.Arg141Term	Late	No	Yes
10 F	c.533C > T	p.Thr178Met	Late	Yes	Yes
11 F	c.914C > G	p.Pro305Arg	Late	No	Yes
12 M	c.604C > T	p.His202Tyr	Late	No	Yes
13 F [27]	c.287C > T	p.Ser96Phe	Late	No	Yes
**14 F**	del814-816GAG*	p.Glu273del*	Late	No	Yes
16 F	c.386 + 1G > A (IVS4 + 1G > A)		Late	Yes	Yes
20 M	c.365A > G	p.Gln122Gly	Late	Yes	Yes
21 M	c.119G > A	p.Arg40His	A, FH	No	Yes
22 M	c.264A > T	p.Lys88Asn	Late	No	Yes
25 M	c.119G > A	p.Arg40His	Late	No	Yes
27 F	c.67C > T	p.Arg23Term	Late	Yes	Yes
32 F [24]	c.386G > A	p.Arg129His	Late	No	Yes
33 F [25]	c.205C > T	p.Gln69Term	Late	Yes	Yes
36 F [25]	delOTC gene >1.6 Mb		Late	Yes	Yes
37 F	c.583G > A	p.Gly195Arg	A, FH	No	Yes
38 F	c.386G > A	p.Arg129His	Late	No	Yes
40 M [27]	c.386 + 5G > A (IVS4 + 5G > A)		Neonatal	No	Yes
**45 F**	c.278C > T*	p.Thr93Ileu*	Late	No	Yes
48 M [24]	c.386G > A	p.Arg129His	Neonatal	No	Yes
49 M	c.86C > T	p.Ala209Val	Late	Yes	Yes
50 F	c.476 T > C	p.Ile159Thr	Late	Yes	Yes
51 M [24]	c.386G > A	p.Arg129His	Late	No	Yes
52 F [25]	c.514A > T	p.Ile172Phe	Late	No	Yes
53 M	c.829C > T	p.Arg277Trp	Late	No	Yes
54 M [26]	c.571C > T	p.Leu191Phe	Late	Yes	Yes
55 M	c.622G > A	p.Ala208Thr	Late	Yes	Yes
**56 M**	c.263A > C*	p.Lys88Thr*	Late	No	Yes
59 M	c.622G > A	p.Ala208Thr	A, FH	No	Yes
**60 F**	c.298 + 1G > C (IVS3 + 1G > C)*		Late	No	Yes
68 F	c.1018C > T	p.Ser340Pro	Late	Yes	Yes
69 F [26]	c.1022C > T	p.Leu341Pro	Late	Yes	Yes
72 F	c.482A > G	p.Asn161Asp	Late	No	Yes
**73 M**	c.144delT*	p.Phe48Leufs*16*	Neonatal	-	No
**74 F**	c.254 T > G*	p.Ileu85Ser*	Late	No	Yes
75 F	c.583G > A	p.Gly195Arg	A, FH	No	Yes
76 M	c.119G > A	p.Arg40His	A, FH	No	Yes
77 M	c.119G > A	p.Arg40His	Late	Yes	Yes
79 F	c.154G > A	p.Glu52Lys	Late	Yes	Yes
80 F	c.583G > A	p.Gly195Arg	Late	Yes	Yes
82 M	c.622G > A	p.Ala208Thr	Late	Yes	Yes
83 M	c.932 T > A	p.Val311Glu	Neonatal	No	Yes
88 F	c.605A > T	p.His202Leu	Late	No	Yes
91 F	c.386 + 2 T > C (IVS4 + 2 T > C)		Late	No	Yes
**94 F**	c.630delA*	p.Lys210Asnfs*20*	Late	Yes	Yes
95 F	delOTC gene >1.6 Mb		Late	Yes	Yes
97 M	c.958C > T	p.Arg320Term	Neonatal	-	No
100 F	c.386G > A	p.Arg129His	Late	No	Yes
103 F [27]	c.211G > T	p.Gly71Term	Late	Yes	Yes

In bold: *mutations not published in the public database HGMD [31].

F: female; M: male.

A: asymptomatic; FH: family history.

Cases 21 and 25, 32 and 51, 37 and 75, 76 and 77 are relatives.

[24-27]: references of primary publications where these patients and their mutations were published.

We include eight new mutations, not previously reported in the public database HGMD® [31]: p.Thr343Arg, p.Glu273del, p.Thr93Ileu, p.Lys88Thr, c.298 + 1G > C (IVS3 + 1G > C), p.Ileu85Ser, p.Phe48Leufs*16, andp.Lys210Asnfs*20. The first six mutations were identified in 2 males and 4 females with late onset and without neurological damage at their last visit. The mutation p.Lys210Asnfs*20 was found in a girl that suffered acute encephalopathy at 6 months of age and has developmental delay in the outcome. Finally, the p.Phe48Leufs*16 mutation was identified in a case with onset two days after birth with ammonia levels of 4584 μmol/L who only survived a few hours.

Table 5 includes the information of the patients with ASS, ASL, CPS and NAGS deficiency . We have no molecular genetic data for any of the cases with argininemia.

**Table 5:** **Mutations identified in patients with ASS, ASL, CPS1 and NAGS deficiencies**

**Patient n°/ [reference]**	**Gene**	**Nucleotide**	**Protein**	**Onset**	**Neurologic damage**
1	ASS	c.256C > T / c.256C > T	p.Arg86Cys / p.Arg86Cys	Late	No
3	ASS	c.206 T > C / c.206 T > C	p.Val69Ala / p.Val69Ala	Neonatal	Yes
**18**	**ASS**	c.557 T > G / c.557 T > G*	p.Met186Arg / p.Met186Arg*	Neonatal	Yes
24	ASS	c.970G > A / c.970G > A	p.Gly324Ser / p.Gly324Ser	Late	No
41	ASS	c.971G > T / c.1168G > A	p.Gly324Val / p.Gly390Arg	Neonatal	Yes
46	ASS	c.1168G > A /c.1168G > A	p.Gly390Arg / p.Gly390Arg	Neonatal	Yes
66 [28]	ASS	c.350G > A / c.1168G > A	p.Gly117Asp / p.Gly390Arg	Late	Yes
**84**	**ASS**	c.970G > A / c.271A > G*	p.Gly324Ser / p.Thr91Ala*	A, NBS	No
85	ASS	c.256C > T / c.256C > T	p.Arg86Cys / p.Arg86Cys	A, NBS	No
86	ASS	c.256C > T / c.256C > T	p.Arg86Cys / p.Arg86Cys	Late	No
96^D^	ASS	c.1168G > A /c.1168G > A	p.Gly390Arg / p.Gly390Arg	Neonatal	-
98 [28]	ASS	c.323G > T + c.356C > T / NF	p.Arg108Leu + p.Thr119Ile / NF	Neonatal	Yes
99	ASS	c.892delG / c.1168G > A	p.Glu298Argfs*/ p.Gly390Arg	Neonatal	Yes
101	ASS	c.919C > T / NF	p.Arg307Cys / NF	A, NBS	No
**9**	**ASL**	c.133 T > A* / c.947G > A*	p.Tyr45Asn* / p.Gly316Glu*	Late	Yes
**29**	**ASL**	c.623C > G / c.623C > G*	p.Pro208Arg / p.Pro208Arg*	Late	Yes
30	ASL	c.623C > G / c.623C > G	p.Pro208Arg / p.Pro208Arg	Late	Yes
39	ASL	c.338G > A / c.338G > A	p.Arg113Gln /p.Arg113Gln	A, Prenatal	Yes
**47**	**ASL**	c.532G > A /c.901G > A*	p.Val178Met / p.Gly301Arg*	Late	Yes
71	ASL	c.1143 + 1G > T / c.1143 + 1G > T	-	Neonatal	Yes
**81**	**ASL**	c.1135C > T/c.1367G > A*	p.Arg379Cys / p.Arg456Gln*	A, NBS	No
**19**	**CPS1**	c.2549G > T*/ NF	p.Arg850Leu* / NF	Late	Yes
**93** [30]	**NAGS**	c.499A > G* / c.916-2A > G*	p.Met167Val */	Late	No

In bold: *Mutations not published in the public database HGMD [31].

A: asymptomatic; NBS: newborn screening; D: Dead in the neonatal period; NF: not found.

Patients 29 and 30 are siblings.

[28,30]: references of primary publications where these patients were published.

Regarding type 1 citrullinemia, twelve different mutations in the ASS1 gene were identified in 14 subjects. The most frequent mutation, p.Gly390Arg, was identified in homozygosis in two patients with neonatal onset and poor outcome. The 3 patients with mutation p.Arg86Cys in homozygosis, diagnosed through NBS, late onset hepatic failure or neurological symptoms, had no neurological sequelae in their last visit. Two new mutations of the ASS1 gene have been identified: p.Met186Arg was identified in homozygosis in one patient of Romanian origin who presented acute encephalopathy “intoxication type” and multiorgan failure in the neonatal period. The other new mutation, p.Thr91Ala, was identified as heterozygous in a case diagnosed by NBS and who remains asymptomatic to date.

Nine mutations in the ASL gene were identified in seven patients (six families) with argininosuccinic aciduria, five of which have not been previously described: p.Tyr45Asn, p.Gly316Glu, p.Pro208Arg, p.Gly301Arg and p.Arg456Gln. All these mutations were found in patients with late onset neurological symptoms, many times without hyperammonemia, except for the p.Arg456Gln mutation which was identified in a patient diagnosed by NBS who remains asymptomatic at 4 years of age.

### Current situation, data at last visit

#### Demographic data

During the 12-month data collection period four patients died, three in the neonatal period (2 OTCD males and 1 ASSD) and one at the age of 9 (CPS1D). Thus, in February of 2013 there were 100 patients registered with UCDs in Spain.

The median age at last visit was 9.96 years (5.29-18). 25% of the patients were over 18 years, 16% were between 12 and 18 years, 33% were between 6-12 years, 22% were between 1-6 year and only 4% of them were under the age of one year.

#### Anthropometric data

Table 6 shows the weight, height, and head circumference for the different age groups at last visit and the BMI for the adult group. The Z-score, calculated according to the tables by Hernández et al. [29] for the Spanish population, are also indicated. The z-score of weight was below -2SD in 4.3% (4/92) of the patients, the z-score of height was below -2SD in 13% (12/89) of the patients and the z-score of head circumference was below -2SD in 20% (5/20) of the patients.

**Table 6:** **Anthropometric data according to age group**

**N = 96**	**0-1 year**	**1-6 years**	**6-12 years**	**12-18 years**	>18 years**
	**(N = 4)**	**(N = 20)**	**(N = 33)**	**(N = 16)**	**(N = 23)**
Age (years)*	0.68 (0.21-0.75)	4.10 (2.98-4.97)	8.61 (7.50-10.2)	15 (14.1-17)	25.3 (21.2-33.3)
Weight (kg)*	8.25 (5.03-8.63)	13.7 (12.8-16.7)	28 (23.9-31.7)	54.1(46.9-66.7)	60 (54.2-75)
Z-score	-0.28	-0.53	-0.008	0.1	0.17
Height (cm)*	67.5 (58.2-70.7)	95 (91-106)	124(120-133)	160 (150-176)	161(153-171)
Z-score	0.71	-0.48	-0.14	-0.06	-0.63
HC (cm)*	43.2 (39.2-43.9)	48.5 (46.5-49.8)	50 (49.5-53)	***	***
Z-score	-0.85	-1.16	-0.68		

*Data reflects median and interquartile range. z-score from tables by Hernández et al [29].

**BMI of adult patients is 23.8 (21.6-27.9).

***No data presented since only observations from 2 patients.

HC: Head circumference.

#### Biochemical data

Table 2 shows some biochemical data at last visit, which are within normal values, except for a slight increase in glutamine.

#### Outcome

Neurological sequelae were seen in 52.5% (53) of the patients. Learning disorders in 50, developmental delay in 33, behavior disorders in 24, epilepsy in 15, motor disorders in 14, and psychiatric disorders in 6. Brain magnetic resonance imaging was performed in 46 patients, 22 of which were normal. Neuropsychological assessment was carried out in 26 patients (13 WISC-IV, 5 McCarthy, 4 Brunet-Lezine, 1 Bayley, 1 Kaufman, 1 Llevant, and 1 Wechsler); the mean intelligence quotient was 87 ± 19. A correlation between the presence of neurological sequelae and the type of disease, type of presentation, liver failure at onset, and ammonia and glutamine levels at diagnosis was seen (Table 7). Neurological outcome was worse in patients with CPS1D and ASLD and in those with neonatal presentation, liver failure at onset and higher levels of ammonia and glutamine at diagnosis.

**Table 7:** **Clinical and biochemical data in relation to neurological outcome**

	**N**	**With neurological impairment % (N)**	**Without neurological impairment % (N)**	**p**
**Disease**				
OTC	65	44.6 (29, 7males)	55.4 (36, 17 males)	0.043*
ASS	21	66.7 (14)	33.4 (7)	
ASL	10	80 (8)	20 (2)	
CPS1	2	100 (2)	0	
ARG1	2	0	2 (100)	
NAGS	1	0	1 (100)	
**Presentation**				
Neonatal	24	75 (18)	25 (6)	0.002*
Late	64	51.6 (33)	48.4 (31)	
Asymptomatic	13	15.4 (2)	84.6 (11)	
**Hepatic failure at diagnosis**				
Yes	17	82.3 (14)	17.7 (3)	0.005*
No	82	45.1 (37)	54.9 (45)	
**Laboratory test at diagnosis**		**Median (IQR)**	**Median (IQR)**	
Ammonia (μmol/L)	92	400 (200-690)	174 (67.2-270)	< 0.001**
Glutamine (μmol/L)	77	1126 (891-1672)	917 (751-1196)	0.019**

Neurological outcome was correlated with the type of UCD disease, type of presentation, presence of liver failure at onset and ammonia and glutamine level at diagnosis. The level of statistical significance was obtained using Pearson chi-square* for qualitative variables and the Mann-Whitney *U* test** for quantitative variables.

Only seven patients (7%) have liver disease, which presents in the form of persistent or intermittent hypertransaminasemia or liver hyper-echogenicity. They are 4 OTCD (3 females) and 3 ASLD patients.

#### Treatment

Dietary treatment was prescribed in 93 patients. Tube feeding was only prescribed in nine cases (8.91%). The patient with NAGS deficiency, the patients that underwent transplantation and two adult cases with OTC deficiency had no dietary restrictions at the time of the study.

In Table 8 the median intake of protein, energy and EAAs in different groups of age is shown and compared to the WHO/FAO/UN recommendations [32] and the UK prescriptions for these patients [33]. Protein and energy intake decreased with age, being slightly below recommendations in the adult group. 50 patients received EAA supplements (20-30% of total protein intake).

**Table 8:** **Diet treatment according to age group**

**Age (N = 93)**	**0-1y (N = 4)**		**1-10y (N = 47)**	**11-18y (N = 20)**	**>18y (N = 22)**
Total proteins (g/kg/day)	1.86 (0.90-2.95)		0.95 (0.7-1.2)	0.84 (0.65-0.94)	0.7 (0.57-0.92)
Energy (calories) (kcal/kg/day)	102 (92.5-140)		72 (58-95)	40 (32-43,5)	36 (30-42)
Essential Aminoacids (g/kg/day)	0.39 (0.2-0.5)		0.3 (0.27-0.43)	0.25 (0.14-0.48)	0.2 (0.11-0.41)
Age	**<6 m**	**6-12 m**	**1-10y**	**11-16y**	**>16y**
WHO/FAO/UNU* (safe levels proteins)	1.77	1.31	0.92-1.14	0.84-0.90	0.84-0.87
UK practice (N = 45)** Proteins	2 (0.7-2.5)	1.6 (1.2-1.8)	1.3 (1-1.7)	0.9 (0.7-1.4)	0.8 (0.4-1.2)

Data reflects median and IQR.

N = number of patients on diet.

y: years; m: months;* Who/FAO/UNU Expert consultation (2007) [32].

**Adam et al. J Hum Nutr Diet 2013; 25: 398-404 [33].

Fifty-eight patients were receiving arginine supplementation (2 CPS1D, 29 OTCD, 18 ASSD, and 9 ASLD) at 156 (109-305) mg/kg/day. Doses were significantly higher in patients with ASSD/ASLD, 165 (120-461) mg/kg/day, in comparison to patients with OTCD/CPS1D, 136 (80-250) (p 0.022). Forty-five patients (44 OTCD and 1 CPS1D) were receiving citrulline at doses of 150 (105-199) mg/kg/day. Twelve of them (11 OTCD and 1 CPS1D) received supplementation of both amino acids, arginine at 115 (55-224) and citrulline at 201 (57-242) mg/kg/day.

63.4% of the patients (64) received nitrogen scavenging drugs (68.2% of the symptomatic patients and 30.8% of the asymptomatic patients), most of them sodium phenylbutyrate (PBA) and a much smaller proportion sodium benzoate or combined treatment (Table 9). The mean age of the patients in treatment with PBA was 10.9 ± 8.3 years and that of patients treated with BZ was 24.2 ± 11.6 years (p < 0.0045). The patient with NAGS deficiency was in treatment with carglumic acid. Other pharmacological treatments received by our UCD patients were: carnitine in 45 patients, vitamin compounds in 25, calcium and vitamin D in 11, and antiepileptic drugs in 12.

**Table 9:** **Pharmacological treatment**

**Drug**	**Patients (N)**	**Mean ± SD (mg/kg/day)**
Sodium phenylbutyrate	50	250 ± 158
Sodium benzoate	4	184 ± 92.3
Combined treatment	10	
Sodium phenylbutyrate		250 ± 134
Sodium benzoate		165 ± 80.5
Carglumic acid	1	15

Liver transplantation was performed in 5 patients, 4 with OTCD (3 females) and 1 with citrullinemia. Two of these patients with OTCD have learning disorders; the rest have no neurological sequelae. There are no food restrictions in their diet and none of the patients receive treatment with nitrogen scavenging drugs. Two patients with OTCD receive 50 and 100 mg/kg/day of citrulline, respectively.

## Discussion

We present the data of the first registry of patients with UCDs in Spain. Since these are rare diseases and there are few patients in each center, most publications on UCDs focus on partial aspects of the disease and only a few describe the natural history of the disease and the current situation of the patients. Recently the data of the American registry [22], a European non-classical UCDs series [34], and the data of the patients in Finland between 1998 and 2007 [6] have been published. The comparison between these series of patients is difficult, particularly due to the differences in the percentage of OTC asymptomatic heterozygous mothers and in the percentage of cases diagnosed by newborn screening. Asymptomatic heterozygous females have not been included in our study and 87.5% of our patients presented symptoms at onset, mostly acute encephalopathy.

Data from 104 cases were collected during one year, 11 of which were diagnosed over this period. As in other UCDs series, the most frequent condition was OTCD (64. 4%, 61.2% females) followed by ASSD and ASLD; the remaining conditions were present in a much lower proportion.

Assuming these were all the cases in Spain in that period, and according to the population data from the Spanish Statistical office [35] (46,609,652 inhabitants and 425,533 newborns), the period prevalence would be 1:448,169 inhabitants and the incidence 1:70,922 newborns. The incidence is lower to that found in other epidemiological studies, e.g., 1:22.179 in the West Midlands in the United Kingdom [5], 1: 39.000 in Finland [6], 1: 41.506 in Italy [4], 1:53.717 in Canada [3] and 1:35.000 in USA [7]. This could be due to the fact that the data was collected for only one year and the age at diagnosis of these diseases is in many cases after the age of one, since OTC deficiency, the most frequent of the UCDs, is not detected by NBS. Furthermore, the expanded NBS is still not performed in all the regions in Spain. Considering that in our series more than half of the cases (55.8%) were diagnosed after 1 year of age, the estimated incidence would be at least double of what we have found.

The median age of our patients at last visit was 9.6 years, 25% of which were over 18 years and 16% were between 12 and 18 years. In the Finnish series [6], the mean age was 13 years, and in European non-classical UCD series [34] the mean age was 18 years (50% of adults). This indicates that there is a need to establish adult centers with specialists for the follow up of this type of patients.

Great heterogeneity was observed regarding the molecular genetic data. A total of 63 different mutations were identified in 98 alleles, 18 (18.3%) of them are newly described in this work (Tables 4 and 5). Six of the eight new mutations in patients with OTC deficiency (p.Thr343Arg, p.Glu273del; p.Thr93Ileu, p.Lys88Thr, p.Ileu85Ser and c.298 + 1G > C) were identified in patients with late onset and no neurological symptoms at last visit, meaning these mutations could be related to a good prognosis, although the gender of the patients (4 females) could have contributed to this good outcome. Conversely, the new and presumably deleterious mutation, p.Phe48Leufs*16, was identified in a severe case of a male with neonatal onset. The most frequent mutation in OTCD was p.Arg129His identified in 5 patients with good neurological outcome. This mutation was described for the first time in 3 of our patients [24]. The second mutation in frequency was p.Arg40His which has already been described in patients with variable phenotype including patients without symptoms [36-38].

None of our patients with type 1 citrullinemia had mutations previously associated with the mild forms of the condition, p.Trp179Arg, p.Val263Met, or p.Gly362Val [39]. Nevertheless, we identified the mutation p.Arg86Cys and the new p.Thr91Ala in four patients with a good neurological outcome, two of them detected by NBS, suggesting these two changes could be related to good prognosis of the disease.

Regarding argininosuccinic aciduria, from the five new mutations identified, p.Arg456Gln was detected in one patient diagnosed by NBS and without neurological damage at the age of four. The other mutation in this patient was p.Arg379Gly, previously associated with a mild phenotype when homozygous [40]. We cannot know if the good neurological evolution of this patient is due to the mild effect of the mutations on the protein function or to the early diagnosis and treatment.

In general, the great genetic heterogeneity in our short series of ASS and ASL deficient patients defies performing a realistic genotype phenotype correlation.

This is the first study that shows anthropometric data in a large series of patients with UCDs. The z-score of the weight and height was in the normal range for Spanish population in all the age groups, although some patients were bellow -2SD (Table 6). The Z-score for the height in the adult group (-0.6) was the lowest, which could be related to the fact that they are the patients with a longer follow-up, but also because during their first years of life they did not benefit from the treatments and nutritional formulas we have today. In this study we have not collected data such as essential fatty acids, vitamins, trace elements, etc., that would inform us of the nutritional status of patients, but we must point out that the levels of albumin are within normal values (Table 2).

As already stated, it is difficult to compare between the series regarding the presence of neurological damage because the inclusion criteria differ and because the establishment of the expanded NBS in certain countries has changed the natural history of the disease for some of these disorders (ASSD, ASLD, and ARG1D), improving their prognosis [41,42]. In our series, that does not include asymptomatic heterozygous mothers and only 12.5% of the cases were diagnosed in a pre-symptomatic state, 52.5% of the cases had neurological complications. In the American series, in which 48% of the OTC patients were asymptomatic heterozygous females, 39% of the patients had psychomotor or intellectual retardation and 35% learning disorders [22]. In the European non-classical UCDs series with 49% asymptomatic cases at diagnosis, 36% had mental retardation or developmental delay [34]. In all studies the disorder with the worst neurological prognosis, is ASLD. In the Finnish series, all ASLD patients that survived had some type of impairment and 30% were epileptic, despite the fact that some had been diagnosed before birth, had early treatment, and an absence of hyperammonemic episodes [6]. In the European series, 65% of the patients with ASLD had psychomotor or intellectual retardation, all those with symptoms at onset, and even three that were diagnosed by NBS [34]. In our series, 80% of the patients with ASL deficiency had neurological damage, even one that was diagnosed in the presymptomatic state. This is indicative that there are other factors besides hyperammonemia causing neurological damage, e.g., high concentrations of argininosuccinic acid in the CNS, low production of nitric oxide etc. The frequency of neurological damage in patients with OTC deficiency was 44.6%, similar to those referred in other series [6,34].

Regarding type 1 citrullinemia, in the European non classical series with 67% of patients diagnosed by NBS, 16% had psychomotor or intellectual retardation [34]. In our series, neurological damage affected 66% of patients, probably because 63.6% of our citrullinemia cases were of neonatal onset and only 13.6% were diagnosed by NBS. Since the expanded NBS has been recently established in our country, these figures might change in the future. Mild variants of citrullinemia, which may possibly be without any clinical significance, have been ascertained in the NBS programs. This is why these programs are being carefully evaluated and citrullinemia has been excluded from the screening panel in some European countries [42].

As in other series, ammonia and glutamine levels at onset were also related to neurological outcome; 75% of the patients without sequelae had ammonia levels ≤270 μmol/L. Thus, to improve neurological prognosis it is necessary to have early diagnosis and treatment of hyperammonemia.

Treatment of UCDs combines dietary and pharmacological therapy. In our series, 92% of the cases were under dietary treatment. A protein restricted diet was used in the European non-classical UCDs series with 82% of the symptomatic patients and 14% of the asymptomatic cases [34], and in the American series with 63% of the total, which includes the asymptomatic heterozygous mothers [22]. The studies describing dietary recommendations for these patients are scarce [11,18,43-45]. The importance of low-protein diet, sufficient caloric intake and supplements of EAA, minerals, vitamins and long-chain polyunsaturated fatty acids in order to prevent deficiencies is pointed out in all the studies. In the European Guidelines [11], recently published, dietary recommendations are based on a C-D evidence level. The Guidelines suggest following the FAO/WHO/UNU recommendations for protein and energy requirements [32], adapting them according to clinical data. They recommend administering 20-30% of the protein (50% in ARG1 deficiency) in the form of EAA in those patients with low protein tolerance in order to maintain growth and metabolic control. During the last few months, two studies have been published regarding dietary treatment in clinical practice in the UK [33] and in other European countries [46] showing that dietary practices vary widely between European IMD centers, particularly with regard to total protein restriction in early infancy, EAAs and approach to nutritional support.

The amount of protein consumed by our patients decreases with age. It is somewhat lower than in the UK [33] but similar to other European countries [46] and according with the WHO recommendations [32], except in the adult group in which is slightly lower (Table 8). In the group of children younger than a year the protein intake is in the high normal range, but we have to say that 3 out of 4 of these patients have citrullinemia diagnosed by NBS, possibly mild forms of the disease. In the rest of the children the intake of protein is in the low normal range of the WHO recommendations. There is no published data on the caloric intake in large series of patients with UCD and therefore we cannot make comparisons with our data, but we can state that our data is in keeping with that obtained through the WHO recommended formulas. In our study 50% of the patients received EAA supplementation, this accounts approximately for 20-30% of the total protein intake. In the UK 30% of the patients received this supplementation and in the rest of Europe 38% on average, with notable differences according to country (100% in Sweden, 64-67% in Germany and Portugal and 24-29% in France, Denmark and Italy). In the USA they are recommended for all patients, supplying 50% of total protein intake in infancy, later reducing this percentage to 25% [45]. The advantage of the EAA would be to decrease the load of ammonia in the urea cycle, since they contain less nitrogen than natural proteins. Besides, given their lower content in tryptophan, tyrosine and phenylalanine, less tryptophan would go to the CNS in situations of hyperammonemia and the production of serotonin would decrease. Lastly, the higher content in branch-chained amino acids would counteract the deficiency of these amino acids found in patients treated with PBA. Therefore, the tendency is to use them in a greater number of patients.

According to the recommendations, arginine and/or citrulline are used to remove ammonia, taking advantage of the urea cycle reactions that are not blocked. In proximal defects, such as in OTC or CPS1 deficiencies, arginine and/or citrulline have been used in 95% (64/67) of patients, arginine alone in 19 cases, citrulline alone in 33 cases, and both in 12 cases. There are no studies comparing the efficacy of the two. For distal defects (ASSD and ASLD) arginine has been used in 87% (27/31) of the cases in significantly higher doses than those used for proximal defects.

The medications used in UCDs are nitrogen scavenging drugs, PBA and SB, which remove the ammonia through alternative routes. In our series, 68% of the symptomatic cases received nitrogen scavenging drugs, a percentage similar to the 66% in the American series [22], and the 72% in the European series [34]. In our series, as in the American one, PBA was used more frequently (49.5%) than SB (3.96%) or the combined therapy (9.9%). In the American series [22], PBA was used in 58% of the cases and SB in 8% of the symptomatic cases. Conversely, in the European series [34], SB was given to 23% of the patients, PBA to 13%, and both to 16%. Furthermore, in our series the age of the patients that were given SB was significantly higher than those to which SB was administered, indicating that the current tendency is to prescribe PBA.

Surprisingly, many patients in our series are still receiving carnitine. Its use was advocated by some in the past, but has proven to be of little benefit in these disorders and not recommended in the last European guidelines.

In summary, in Spain there are currently 100 living patients registered with UCDs, of which 87% were index-patients diagnosed due to symptoms. 25% have reached adulthood and 52% have neurological damage. 92% of the patients are under nutritional therapy, consisting of a normocaloric, low-protein diet, supplemented with EAA in 50% of the cases. 87% of the cases with ASS/ASL deficiency receive treatment with arginine, and 98% of the patients with OTC/CPS1 deficiency receive arginine and/or citrulline. 68% of the symptomatic cases are treated with a nitrogen scavenging drug, mainly sodium phenylbutyrate. The Z-score of the weight and height of our patients was normal in all age groups, although lower than the median in height, our study being the first to show the anthropometric data in these patients.

## Conclusions

Expanded registries of rare diseases are indispensable to help understand the natural history of these pathologies and how they may change over time due to better diagnostic and therapeutic tools. It also helps understand the pathophysiology of these diseases, as many genetic or phenotypic data may not be reported individually. In our case, we report 18 novel mutations for various deficiencies.

In order to improve the neurological prognosis of these patients, the expanded newborn screening should be extended to the entire country. As well as this, there is a need for greater diffusion of the knowledge regarding these diseases to general pediatricians, neurologists and neonatologists so we can diagnose them in the pre-symptomatic state or when ammonia is below the levels that cause irreversible neurological damage.

There is a clear need to incorporate specialists who treat adults with these diseases in metabolic units, for the follow up and treatment of patients once they reach adulthood. Likewise, the integration of dietitians, neuropsychologists and social workers in metabolic units are also required to facilitate the multiple needs these patients present.

## Availability of supporting data

OMIM: http://www.ncbi.nlm.nih.gov/omim/

OMIM is a comprehensive, authoritative compendium of human genes and genetic phenotypes that is freely available and updated daily.

HGMD: http://www.biobase-international.com/product/hgmd

HGMD^®^ Professional is a unique resource providing comprehensive data on human inherited disease mutations to genetics and genomic research. Its compilation enables quick access to both single mutation queries and advanced search applications.

## Abbreviations

UCDs: Urea cycle disorders
NAGSD: N-acetylglutamate synthase deficiency
CPS1D: Carbamoylphosphate synthetase 1 deficiency
OTCD: Ornithine transcarbamylase deficiency
ASSD: Argininosuccinate synthetase deficiency
ASLD: Argininosuccinate lyase deficiency
ARG1D: Arginase 1 deficiency
HHH: Hyperornithinemia-hyperammonemia-homocitrullinuria
EAA: Essential amino acid
BZ: Sodium benzoate
PBA: Sodium phenylbutyrate
NBS: Newborn screening
AECOM: Spanish Association of Inborn Errors of Metabolism
IMD: Inherited metabolic diseases
SD: Standard deviation
IQR: Interquartile range
CNS: Central Nervous System

## References

[1] Brusilow SW (1984). Arginine, an indispensable amino acid for patients with inborn errors of urea synthesis. J Clin Invest.

[2] Brusilow S, Horwich A, Scriver C, Beaudet A, Sly W, Valle D (2001). Urea cycle enzymes. The Metabolic and Molecular Bases of Inherited Diseases.

[3] Applegarth DA, Toone JR, Lowry RB (2000). Incidence of inborn errors of metabolism in British Columbia, 1969-1996. Pediatrics.

[4] Dionisi-Vici C, Rizzo C, Burlina AB, Caruso U, Sabetta G, Uziel G, Abeni D (2001). Inborn errors of metabolism in the Italian pediatric population. J Pediatr.

[5] Sanderson S, Green A, Perece M, Burton H (2006). The frequency of inherited metabolic disorders in the west midlands, United Kigdom. Arch Dis Child.

[6] Keskinen P, Siitonen A, Salo M (2008). Hereditary urea cycle diseases in Finland. Acta Paediatr.

[7] Summar ML, Stefan Koelker S, Freedenberg D, Le Mons C, Haberle J, Lee HS, Kirmse B, the European Registry and Network for Intoxication Type Metabolic Diseases (E-IMD), the Members of the Urea Cycle Disorders Consortium (UCDC) (2013). The incidence of urea cycle disorders. Mol Genet Metab.

[8] Saudubray JM, Nassogne MC, de Lonlay P, Touati G (2002). Clinical approach to inherited metabolic disorders in neonates: an overview. Semin Neonatol.

[9] Summar ML, Dobbelaere D, Brusilow S, Lee B (2008). Diagnosis, symptoms frequency and mortality of 260 patients with urea cycle disorders from a 21-year multicenter study of acute hyperammonaemic episodes. Acta Paediatr.

[10] Wijburg FA, Nassogne MC, Saudubray JM, van den Berghe G, Walter JH (2012). Disorders of the urea cycle and related enzymes. Inborn Metabolic Diseases, Diagnosis and Treatment.

[11] Häberle J, Boddaert N, Burlina A, Chakrapani A, Dixon M, Huemer M, Karall D, Martinelli D, Crespo PS, Santer R, Servais A, Valayannopoulos V, Lindner M, Rubio V, Dionisi-Vici C (2012). Suggested guidelines for the diagnosis and management of urea cycle disorders. Orphanet J Rare Dis.

[12] Sander J, Janzen N, Sander S, Steuerwald U, Das AM, Scholl S, Trefz FK, Koch HG, Haberle J, Korall H, Marquardt I, Korenke C (2003). Neonatal screening for citrullinaemia. Eur J Pediatr.

[13] Mercimek-Mahmutoglu S, Moeslinger D, Häberle J, Engel K, Herle M, Strobl MW, Scheibenreiter, Muehl A, Stöckler-Ipsiroglu (2010). Long-term outcome of patients with argininosuccinate lyase deficiency diagnosed by newborn screening in Austria. Mol Genet Metab.

[14] Summar M (2001). Current strategies for the management of neonatal urea cycle disorders. J Pediatr.

[15] Couce ML, Bustos G, García-Alixc A, Lázaro A, Martínez-Pardo M, Molina A, Saénz de Pipaón M, Serrano M, Sanjurjo P (2009). Guía clínica de diagnóstico y tratamiento urgente de hiperamonemia neonatal. An Pediatr (Barc).

[16] Schaefer F, Straube E, Oh J, Mehls O, Mayatepek E (1999). Dialysis in neonates with inborn errors of metabolism. Nephrol Dial Transplant.

[17] Picca S, Dionisi-Vici C, Abeni D, Pastore A, Rizzo C, Orzalesi M, Sabetta G, Rizzoni G, Bartuli A (2001). Extracorporeal dialysis in neonatal hyperammonemia: modalities and prognostic indicators. Pediatr Nephrol.

[18] Scaglia F (2010). New insights in nutritional management and amino acid supplementation in urea cycle disorders. Mol Genet Metab.

[19] Enns GM, Berry SA, Berry GT, Rhead WJ, Brusilow SW, Hamosh A (2007). Survival after treatment with phenylacetate and benzoate for urea-cycle disorders. N Engl J Med.

[20] Stevenson T, Millan MT, Wayman K, Berquist WE, Sarwal M, Johnston EE, Esquivel CO, Enns GM (2009). Long-term outcome following pediatric liver transplantation for metabolic disorders. Pediatr Transplant.

[21] Kim IK, Niemi AK, Krueger C, Bonham CA, Concepcion W, Cowan TM, Enns GM, Esquivel CO (2013). Liver transplantation for urea cycle disorders in pediatric patients: a single-center experience. Pediatr Transplant.

[22] Tuchman M, Lee B, Lichter-Konecki U, Summar ML, Yudkoff M, Cederbaum SD, Kerr DS, Diaz GA, Seashore MR, Lee HS, McCarter RJ, Krischer JP, Batshaw ML, Urea Cycle Disorders Consortium of the Rare Diseases Clinical Research Network (2008). Cross-sectional multicenter study of patients with urea cycle disorders in the United States. Mol Genet Metab.

[23] Kölker S, Dobbelaere D, Chaprani A, Parker S, Burgard P, Hoffmann G, De Baere L, Stroobant N, Haeberle J, Baumgartner M (2011). European registry and network for intoxication type metabolic diseases (E-IMD)(Abstract). J Inher Matabolic Dis.

[24] García-Pérez MA, Sanjurjo P, Rubio V (1995). Demonstration of the spf-ash mutation in Spanish patients with ornithine transcarbamylase deficiency of moderate severity. Hum Genet.

[25] Climent C, García-Pérez MA, Sanjurjo P, Ruiz-Sanz JI, Vilaseca MA, Pineda M, Campistol J, Rubio V (1999). Identification of a cytogenetic deletion and of four novel mutations (Q69X, I172F, G188V, G197R) affecting the gene for ornithine transcarbamylase (OTC) in Spanish patients with OTC deficiency. Hum Mutat.

[26] Climent C, Rubio V (2002). Identification of seven novel missense mutations, two splice-site mutations, two microdeletions and a polymorphic amino acid substitution in the gene for ornithine transcarbamylase (OTC) in patients with OTC deficiency. Hum Mutat.

[27] Arranz JA, Riudor E, Marco-Marín C, Rubio V (2007). Estimation of the total number of disease-causing mutations in ornithine transcarbamylase (OTC) deficiency. Value of the OTC structure in predicting a mutation pathogenic potential. J Inherit Metab Dis.

[28] Vilaseca MA, Kobayashi K, Briones P, Lambruschini N, Campistol J, Tabata A, Alomar A, Rodès M, Lluch M, Saheki T (2001). Phenotype and genotype heterogeneity in Mediterranean citrullinemia. Mol Genet Metab.

[29] Hernández M, Castellet J, Narvaiza JL, Rincón JM, Ruiz I, Sánchez E, Sobradillo B, Zurimendi A (2002). Curvas y tablas de crecimiento. Instituto de Investigación sobre Crecimiento y Desarrollo. Fundación F. Orbegozo. Bilbao. 3ª Edición.

[30] Bélanger-Quintana A, Martínez-Pardo M, García MJ, Wermuth B, Torres J, Pallarés E, Ugarte M (2003). Hyperammonaemia as a cause of psicosis in an adolescent. Eur J Pediatr.

[31] Stenson PD, Mort M, Ball EV, Shaw K, Phillips AD, Cooper DN (2014). HMGD professional The Human Gene Mutation Database: building a comprehensive mutation repository for clinical and molecular genetics, diagnostic testing and personalized genomic medicine. Hum Genet.

[32] Who/FAO/UNU Expert consultation (2007). Protein and Amino Acid Requirements in Human Nutrition, WHO Technical Reports Series 935.

[33] Adam S, Champion H, Daly A, Dawson S, Dixon M, Dunlop C, Eardley J, Evans S, Ferguson C, Jankowski C, Lowry S, MacDonald A, Maritz C, Micciche A, Robertson L, Stafford J, Terry A, Thom R, van Wyk K, Webster D, White FJ, Wildgoose J, British Inherited Metabolic Diseases Group (BIMDG) Dietitian’s Group (2012). Dietary management of urea cycle disorders: UK practice. J Hum Nutr Diet.

[34] Rüegger CM, Lindner M, Ballhausen D, Baumgartner MR, Beblo S, Das A, Gautschi M, Glahn EM, Grünert SC, Hennermann J, Hochuli M, Huemer M, Karall D, Kölker S, Lachmann RH, Lotz-Havla A, Möslinger D, Nuoffer JM, Plecko B, Rutsch F, Santer R, Spiekerkoetter U, Staufner C, Stricker T, Wijburg FA, Williams M, Burgard P, Häberle J (2014). Cross-sectional observational study of 208 patients with non-classical urea cycle disorders. J Inherit Metab Dis.

[35] **Instituto Nacional de Estadística. **http://www.ine.es/.

[36] Tuchman M, Morizono H, Rajagopal BS, Plante RJ, Allewell NM (1998). The biochemical and molecular spectrum of ornitine transcarbamylase deficiency. J Inherit Metab Dis.

[37] Tuchman M, Jaleel N, Morizono H, Sheeby L, Lynch MG (2002). Mutations and polymorphisms in the human ornithine transcarbamylase (OTC) gene. Hum Mutat.

[38] Mavinakere M, Morizono H, Shi D, Alewell NM, Tuchman M (2001). The clinically variable R40H mutant ornithine carbamoyltransferase shows cytosolic degradation of the precursor protein in CHO cells. J Inherit Metab Dis.

[39] Haberle J, Pauli S, Linnebank M, Kleijer WJ, Bakker HD, Wanders RJA, Harms E, Koch HG (2002). Structure of the human argininosuccinate synthetase gene and an improved system for molecular diagnostics in patients with classical and mild citrullinemia. Hum Genet.

[40] Kleijer WJ, Garritsen VH, Linnebank M, Mooyer P, Huijmans JG, Mustonen A, Simola KO, Arslan-Kirchner M, Battini R, Briones P, Cardo E, Mandel H, Tschiedel E, Wanders RJ, Koch HG (2002). Clinical, enzymatic and molecular genetic characterization of a biochemical variant type of argininosuccinic aciduria: prenatal and postnatal diagnosis in 5 unrelated families. J Inherit Metab Dis.

[41] Ficicioglu C, Mandell R, Shih VE (2009). Arginosuccinate lyase deficiency: longterm outcome of 13 patients detected by newborn screening. Mol Genet Metab.

[42] Lindner M, Gramer G, Haege G, Fang-Hoffmann J, Schwab KO2, Tacke U, Trefz FK, Mengel E, Wendel U, Leichsenring M, Burgard P, Hoffmann JF (2011). Efficacy and outcome of expanded newborn screening for metabolic diseases - Report of 10 years from South-West Germany. Orphanet J Rare Dis.

[43] Urea cycle Disorders Conference Group (2001). Consensus statement from a conference for the management of patients with urea cycle disorders. J Pediatr.

[44] Acosta PB, Yanicelli S, Ryan AS, Arnold G, Marriage BJ, Plewinska M, Bernstein L, Fox J, Lewis V, Miller M, Velazquez A (2005). Nutritional therapy improves growth and protein status of children with urea cycle enzyme defect. Mol Genet Metab.

[45] Singh RH (2007). Nutritional management of patients with urea cycle disorders. J Inherit Metab Dis.

[46] Adam S, Almeida MF, Assoun M, Baruteau J, Bernabei SM, Bigot S, Champion H, Daly A, Dassy M, Dawson S, Dixon M, Dokoupil K, Dubois S, Dunlop C, Evans S, Eyskens F, Faria A, Favre E, Ferguson C, Goncalves C, Gribben J, Heddrich-Ellerbrok M, Jankowski C, Janssen-Regelink R, Jouault C, Laguerre C, Le Verge S, Link R, Lowry S, Luyten K (2013). Dietary management of urea cycle disorders: European practice. Mol Genet Metab.

